# Biomarkers for Predicting Anti-Programmed Cell Death-1 Antibody Treatment Effects in Head and Neck Cancer

**DOI:** 10.3390/curroncol30060410

**Published:** 2023-06-02

**Authors:** Katsunori Tanaka, Hitoshi Hirakawa, Mikio Suzuki, Teruyuki Higa, Shinya Agena, Narumi Hasegawa, Junko Kawakami, Masatomo Toyama, Tomoyo Higa, Hidetoshi Kinjyo, Norimoto Kise, Shunsuke Kondo, Hiroyuki Maeda, Taro Ikegami

**Affiliations:** Department of Otorhinolaryngology, Head and Neck Surgery, Graduate School of Medicine, University of the Ryukyus, 207 Uehara, Nishihara-cho, Nakagami-gun, Okinawa 903-0215, Japan; mizuki0415@gmail.com (K.T.); aoi23@med.u-ryukyu.ac.jp (H.H.); tellurteru@yahoo.co.jp (T.H.); harugen3@yahoo.co.jp (S.A.); puyoraer99110@gmail.com (M.T.); tomoyo_12_26@hotmail.co.jp (T.H.); hidechanman223@yahoo.co.jp (H.K.); norimoto7@gmail.com (N.K.); kouhouiinn@yahoo.co.jp (S.K.); maeidahiroyuki@yahoo.co.jp (H.M.); ikegami@med.u-ryukyu.ac.jp (T.I.)

**Keywords:** anti-programmed cell death-1 antibody, programmed cell death ligand-1, polymorphism, nutrition, head and neck cancer, immune-related adverse events, disease prognosis

## Abstract

In recurrent or metastatic head and neck squamous cell carcinoma (R/M-HNSCC), survival outcomes are significantly better in patients who receive anti-programmed cell death-1 (PD-1) monoclonal antibody therapy than in those who receive standard therapy. However, there is no established biomarker that can predict the anti-PD-1 antibody treatment effect and immune-related adverse events (irAEs) in these patients. This study investigated the inflammatory and nutritional status in 42 patients with R/M-HNSCC and programmed cell death ligand-1 (PD-L1) polymorphisms (rs4143815 and rs2282055) in 35 of the 42 patients. The 1- and 2-year overall survival was 59.5% and 28.6%, respectively; the 1- and 2-year first progression-free survival was 19.0% and 9.5%, respectively, and the respective second progression-free survival was 50% and 27.8%. Performance status and inflammatory and nutritional status (assessed by the geriatric nutritional risk index, modified Glasgow prognostic score, and prognostic nutritional index) were identified as significant indicators of survival outcomes in multivariate analysis. Patients with ancestral alleles in PD-L1 polymorphisms had less frequent irAEs. Performance status and inflammatory and nutritional status before treatment were closely related to survival outcomes after PD-1 therapy. These indicators can be calculated using routine laboratory data. PD-L1 polymorphisms may be biomarkers for predicting irAEs in patients receiving anti-PD-1 therapy.

## 1. Introduction

The Global Cancer Observatory estimated the number of patients with head and neck cancer to be approximately 0.93 million worldwide in 2020 [1]. According to monitoring of cancer incidence in Japan, the estimated 5-year relative survival rate was 63.5% in patients diagnosed with oral or pharyngeal cancer between 1993 and 1999 in Japan [2].

Anti-programmed cell death-1 (anti-PD-1) monoclonal antibodies are immune checkpoint inhibitors (ICIs) recently introduced to treat recurrent or metastatic head and neck squamous cell carcinoma (R/M-HNSCC) [3,4]. Overall survival (OS) was found to be significantly longer (hazard ratio 0.7), and the 1-year OS rate (36.0%) was 19% higher in patients with R/M-HNSCC who had received the anti-PD-1 antibody nivolumab than in those who had received standard therapy [3]. Furthermore, nivolumab tripled the estimated 24-month OS rate (16.9%), regardless of tumor programmed cell death ligand-1 (PD-L1) expression [4]. Clinical trials using ICIs have now expanded to include induction treatment aimed at organ preservation and improving survival [5].

Nivolumab is approved in Japan for the treatment of platinum-resistant or platinum-intolerant R/M-HNSCC. Real-world data from a multicenter retrospective study in Japan revealed a median OS of 9.5 months, a 1-year OS of 43.2%, and an objective response rate (ORR) of 15.7% [6]. However, some tumors progress rapidly on anti-PD-1 treatment, a phenomenon known as hyperprogression, and not all patients can tolerate nivolumab because of immune-related adverse events (irAEs). Grade ≥ 3 irAEs occurred in 5.9% of patients in a Japanese real-world study [6] and in 13.1% of those in a randomized controlled trial [3]. Although PD-L1 expression in tumors and surrounding immune cells has been proposed as the reason for the treatment effect, this finding has not been validated in patients with HNC [7]. Therefore, there is a need for further research to identify biomarkers that can predict the anti-PD-1 antibody treatment effects and the possible occurrence of irAEs before the administration of nivolumab to these patients. 

Previous reports suggest that polymorphisms in PD-1 and PD-L1 are associated with a higher risk of gastric, bladder, and hepatocellular cancers [8], irAEs [9], better survival outcomes in non-small cell lung cancer (NSCLC) [10] and prostate cancer [11], and extended clinical benefit in NSCLC [12]. However, there is limited information on the relationship between PD-1/PD-L1 polymorphisms and survival outcomes in patients with HNSCC [13]. There are several candidate PD-1/PD-L1 single nucleotide polymorphisms (SNPs) that could be used to predict ICI treatment effects and the occurrence of irAEs. The rs4143815 SNP in the 3′-untranslated region of PD-L1 is a structural polymorphism. MicroRNAs can bind to the mRNA of target genes to degrade the mRNAs or to prevent their translation through epigenetic regulation [14]. The rs4143815 SNP is located in the miR-570 binding site, and rs4143815 C/C alleles induce overexpression of PD-L1 [15,16]. In NSCLC, prognosis after ICI therapy is poorer in patients with rs4143815 G/G (ancestral) alleles compared with other alleles [10,12]. Similarly, patients with the rs4143815 G allele are reported to have an increased risk of type I diabetes mellitus [17]. The rs2282055 SNP is in the PD-L1 intron and is known to affect the therapeutic effect of nivolumab in NSCLC [10], although the mechanism remains unclear. Given that the frequencies of these two alleles in rs2282055 and rs4143815 vary markedly according to region, it is possible that anti-PD-1 antibody therapeutic effects vary according to ethnicity. However, no previous study has analyzed these SNPs and their therapeutic effects in HNSCC.

Systemic inflammation and nutritional status [18,19,20] have been associated with cancer prognosis. Many indicators of inflammation, including the neutrophil-to-lymphocyte ratio (NLR), are useful biomarkers for predicting the therapeutic effect and prognosis in HNC cancer [21,22,23,24]. A recent meta-analysis demonstrated that low pretreatment nutritional status is correlated with survival outcomes [20]. However, there are few relevant clinical studies concerning ICI therapy, and more are needed.

The aim of this study was to clarify the ICI treatment effect in patients with R/M-HNSCC. For this purpose, the PD-L1 polymorphisms (rs4143815 and rs2282055) and nutritional status were selected as possible biomarkers for anti-PD-1 antibody treatment effects and the occurrence of irAEs.

## 2. Materials and Methods

This study included (1) a retrospective clinical review of the medical charts of consecutive patients treated with nivolumab and (2) PD-L1 experiments in which we analyzed the immunohistochemical expression of PD-L1 and SNPs of PD-L1 (rs4143815 and rs2282055) in consenting patients.

Patients were considered refractory to platinum if they relapsed within 6 months of receiving a platinum-based regimen. Consecutive patients with platinum-resistant or platinum-intolerant R/M-HNSCC were selected as candidates for treatment with nivolumab between 1 April 2017 and 31 December 2020 at the University of the Ryukyus Hospital. The observation period ended on 31 December 2022. Blood samples were obtained to investigate SNPs. Various protocols were used after the administration of nivolumab as a second or later line of treatment according to the condition of the individual patient.

The study was conducted with the approval of the Institutional Review Board of the University of Ryukyus (project identification codes: 156 and 1860) in accordance with the 1975 Declaration of Helsinki, as revised in 2008. Written informed consent was obtained from all participants before collection of blood samples.

Nivolumab was administered intravenously at a dose of 3 mg/kg at 2-week intervals. In September 2018, the maintenance dose was changed to 240 mg in Japan. Thus, from September 2020, nivolumab was administered at initial and subsequent doses of 240 mg every 2 weeks or 480 mg every 4 weeks according to the needs of each patient. For counting the number of administered doses of nivolumab, a dose of 480 mg was counted as two doses. Where nivolumab was discontinued because of irAEs or disease progression, subsequent lines of chemotherapy were administered at the physician’s discretion.

### 2.1. Retrospective Clinical Review of Medical Charts

Medical charts were retrospectively reviewed to obtain clinical data, including primary treatments and inflammatory and nutritional parameters before administration of nivolumab as well as nivolumab-related adverse events in individual patients.

The geriatric nutritional risk index (GNRI) [18], modified Glasgow prognostic score (mGPS) [19], Prognostic Nutritional Index (PNI) [20], NLR [25], and Eastern Cooperative Oncology Group performance status (PS) [26,27] were used to estimate nutritional status before administration of nivolumab. These scores have been used previously to evaluate the inflammatory and nutritional status in cancer patients. PNI is calculated from the serum albumin level and lymphocyte count [28]. The NLR is obtained by dividing the absolute neutrophil count by the absolute lymphocyte count and was evaluated twice, once at the start of nivolumab administration and 8 weeks later.

During the observation period, information on each patient’s clinicopathologic parameters and treatment outcome was recorded at least every 4 weeks for the first year and every 2–3 months for 2–5 years, according to the patient’s condition.

Overall survival (OS), first progression-free survival (PFS), and second progression-free survival (PFS-2) were investigated as prognostic indicators. The starting point for OS, PFS, and PFS-2 was defined as the day of the first nivolumab administration. The final prognosis was assessed on 31 December 2022. OS was defined as the interval between the date of the first nivolumab administration and the date of death from any cause or 31 December 2022, whichever came first. PFS was defined as the interval between the date of the first nivolumab administration and the first tumor progression (defined by the Response Evaluation Criteria in Solid Tumors guideline, version 1.1) or 31 December 2022, whichever came first. PFS-2 was defined as the interval between the date of the first nivolumab administration and either death, the second progression on subsequent treatment, or 31 December 2022, whichever occurred first, and was analyzed in patients who received other treatments after cessation of nivolumab because of tumor progression or intolerance to nivolumab.

The duration of nivolumab administration, total dose of nivolumab, and best overall response were also recorded. Adverse events were assessed using the Common Terminology Criteria for Adverse Events, version 5.0, in consultation with other specialties.

Survival curves were estimated using the Kaplan–Meier method and compared between groups using the log-rank test. The prognostic significance of variables related to OS, PFS, and PFS-2 was assessed in multivariate analysis using a Cox proportional-hazards model. The ORR was the sum of the complete and partial responses. The disease control rate (DCR) was the sum of the complete response, partial response, and stable disease. All analyses were performed using the SPSS statistical package (SPSS for Windows, version 25.0; SPSS, Inc., Chicago, IL, USA). All tests were two-sided, and *p*-values < 0.05 were considered statistically significant.

### 2.2. Immunohistochemical Analysis of PD-L1 Expression and PD-L1 SNPs (rs4143815 and rs2282055)

**Figure 1:**
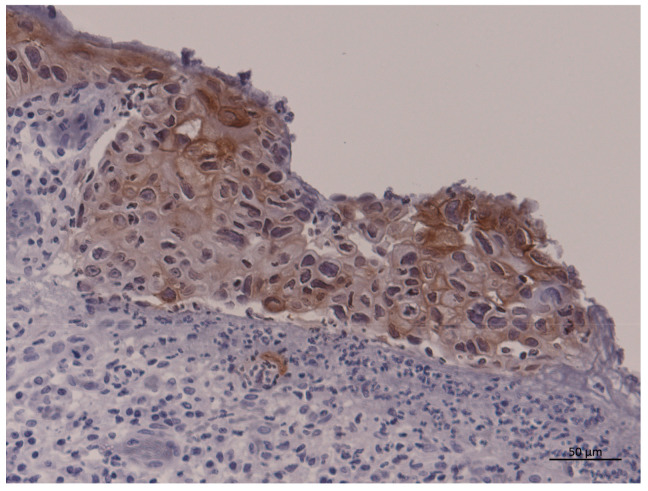
Representative immunohistochemistry for PD-L1. Cell membranes in tumor cells were stained but some mononuclear cells around the tumor also showed PD-L1 expression in this case. Bar = 50 μm. PD-L1, programmed cell death ligand-1.

Four-micrometer-thick sections from paraffin-embedded block samples obtained from primary lesions before starting primary treatment were deparaffinized in xylene and rehydrated in a graded series of alcohol. Epitope retrieval was achieved by heating to 100 °C for 10 min in 1 mM EDTA buffer (pH 8.0). Endogenous peroxidase activity was quenched by incubating the sections in 0.3% H_2_O_2_ in methanol for 20 min at room temperature. A SAB-PO Kit (Nichirei Biosciences Inc., Tokyo, Japan) was used to detect immunoreactivity to PD-L1 according to the manufacturer’s protocol. After blocking non-specific reactions with 10% goat serum, the sections were incubated with primary antibodies for 1 h at room temperature. A rabbit monoclonal anti-PD-L1 antibody (E1L3N^®^, Cell Signaling Technology, Danvers, MA, USA) was used at a 1:200 dilution with Protein Block Serum-Free (Dako; Agilent Technologies, Inc., Santa Clara, CA, USA). Positive PD-L1 expression was defined as a stained cell membrane by immunohistochemistry (Figure 1).

### 2.3. Measurement of PD-L1 SNPs

Peripheral blood samples were collected into EDTA-containing tubes and centrifuged at 2000× *g* for 15 min. The obtained buffy coat was stored at −80 °C until DNA extraction. The genomic DNA was extracted from the buffy coat using a Maxwell 16 Blood DNA Purification Kit (Promega, Madison, WI, USA) and stored at −20 °C. The rs2282055 in the PD-L1 intron 1 and rs4143815 in the 3′-untranslated region of PD-L1 were analyzed by real-time quantitative polymerase chain reaction (PCR) using the Taqman™ probe method. The TaqMan SNP genotyping assay was used for rs2282055 (assay ID C_1409286_1, catalog number 4351379, Applied Biosystems, Waltham, MA, USA) and rs4143815 (assay ID C_31941235_10, catalog number 4351379, Applied Biosystems). Real-time PCR was performed using the CFX96 Touch™ Real-Time PCR Detection System (BioRad, Hercules, CA, USA) and the TaqMan genotyping assay (Applied Biosystems) according to the manufacturer’s instructions. The PCR profile was as follows: 50 °C for 2 min and 95 °C for 10 min followed by 40 cycles of 95 °C for 15 s and 60 °C for 1 min.

## 3. Results

### 3.1. Patient Characteristics

**Figure 2:**
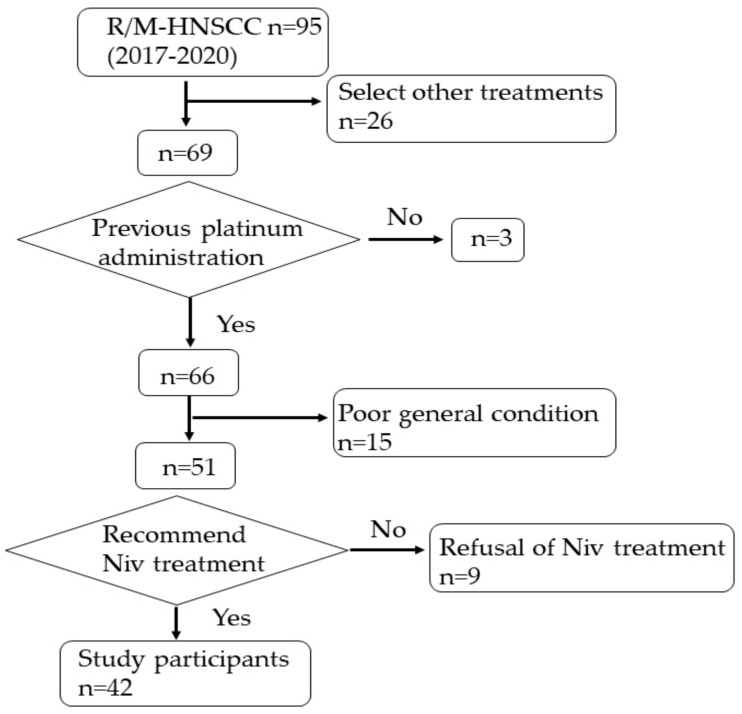
Flowchart of patient selection.

There were 95 patients with R/M-HNSCC at the University of the Ryukyus Hospital between 1 April 2017 and 31 December 2020. Of these, 51 patients were recommended to receive nivolumab treatment (Figure 2). Nine patients declined nivolumab treatment, leaving 42 patients with R/M-HNSCC for analysis in this study. The pathologic diagnosis was squamous cell carcinoma in all cases. 

The patient characteristics are described in detail in Table 1. Briefly, there were 36 men and 6 women with a mean age of 60.5 years (range 26–81 years). The primary lesions were in the hypopharynx (*n* = 14), oropharynx (*n* = 13), oral cavity (*n* = 9), nasopharynx (*n* = 2), or other sites (*n* = 4). Seven of the 13 cases of oropharyngeal squamous cell carcinoma were human papillomavirus-positive. At the time of the first nivolumab administration, the PS was 0 in 29 cases, 1 in 10 cases, and ≥2 in 3 cases. 

The number of nivolumab doses ranged from 1 to 74, with a mean of 13.6 and a median of 9.5. When compared by PS, the mean number of nivolumab doses was 14.0 for PS 0 or 1 and 8.3 for PS ≥ 2 (*p* < 0.61, Mann–Whitney *U* test). Nivolumab was administered as a first-line treatment after recurrence in 12 patients, as a second-line treatment in 14 patients, and as a third-line or later treatment in 16 patients. At the start of nivolumab administration, 14 patients had primary recurrence, 10 had regional lymph node recurrence, and 31 had distant metastasis. The distant metastases were in the lung (*n* = 23), distant lymph nodes (*n* = 10), bone (*n* = 7), and skin, liver, or heart (*n* = 1 each).

Nivolumab was continued in one patient who achieved a long complete remission. Eighteen patients were switched from nivolumab to other treatments. Several treatment protocols were used after nivolumab administration depending on the patient’s condition. The paclitaxel (PTX)–Cet protocol [29] was frequently selected after nivolumab administration (Table 2). Twenty-three patients did not receive further treatment after nivolumab because of progressive disease or poor general condition (*n* = 22) or due to the achievement of a complete response (*n* = 1) (Table 2). There were no cases of pseudoprogression in the present study.

### 3.2. Survival Analysis

**Figure 3:**
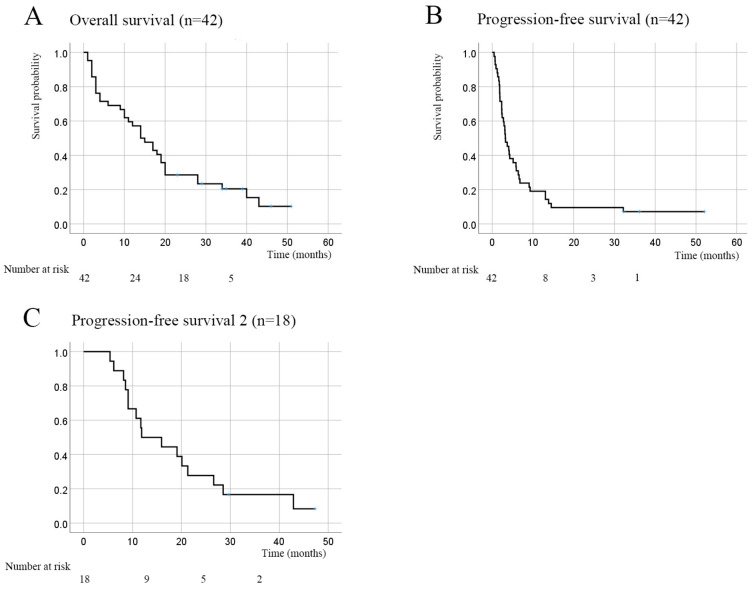
Kaplan–Meier curves for overall survival, progression-free survival, and progression-free survival-2. (**A**) Overall survival (OS; *n* = 42). The OS rate was 59.5% at 1 year and 28.6% at 2 years, with a median OS of 15 months. (**B**) Progression-free survival (PFS; *n* = 42). The PFS rate was 19.0% at 1 year and 9.5% at 2 years, with a median PFS of 3.2 months. (**C**) Second progression-free survival (PFS-2; *n* = 18). The PFS-2 rate was 50% at 1 year and 27.8% at 2 years, with a median PFS-2 of 11.9 months.

The OS rate for all 42 cases was 59.5% at 1 year and 28.6% at 2 years, with a median OS of 15 months (Figure 3A). The PFS rate was 19.0% at 1 year and 9.5% at 2 years, with a median PFS of 3.2 months (Figure 3B). The PFS-2 rate was 50% at 1 year and 27.8% at 2 years, with a median PFS-2 of 11.9 months (Figure 3C). Three patients (7.2%) achieved a complete response, 4 (9.5%) achieved a partial response, 14 (33.3%) had stable disease, and 21 had progressive disease. Thus, the ORR was 16.7%, and the DCR was 50.0% (Table 3). Eighteen patients received subsequent treatments after nivolumab (PTX + Cet, *n* = 15); 5-fluorouracil + cisplatin + pembrolizumab, *n* = 2; tegafur–gimestat–otastat potassium, *n* = 1). One of these patients had a complete response, three had a partial response, seven had stable disease, and seven had progressive disease. The OS was significantly better in patients with PS 0 or 1 than in those with PS ≥ 2 (*p* = 0.048). Patients who received 4 or more doses of nivolumab had a better OS (*p* < 0.001, Table 4).

### 3.3. Nutritional Status

Mean and median NLR values at the start of treatment with nivolumab were 6.1 and 4.9, respectively. There was no significant difference in OS according to whether the NLR was higher or lower than 4.9 (*p* = 0.22). However, OS was significantly better in patients with an NLR lower than 4.9 at 8 weeks after starting nivolumab (*p* = 0.029).

Six patients received enteral nutrition via a nasogastric or percutaneous endoscopic gastrostomy tube. There was no significant difference in survival time according to enteral nutrition status.

**Figure 4:**
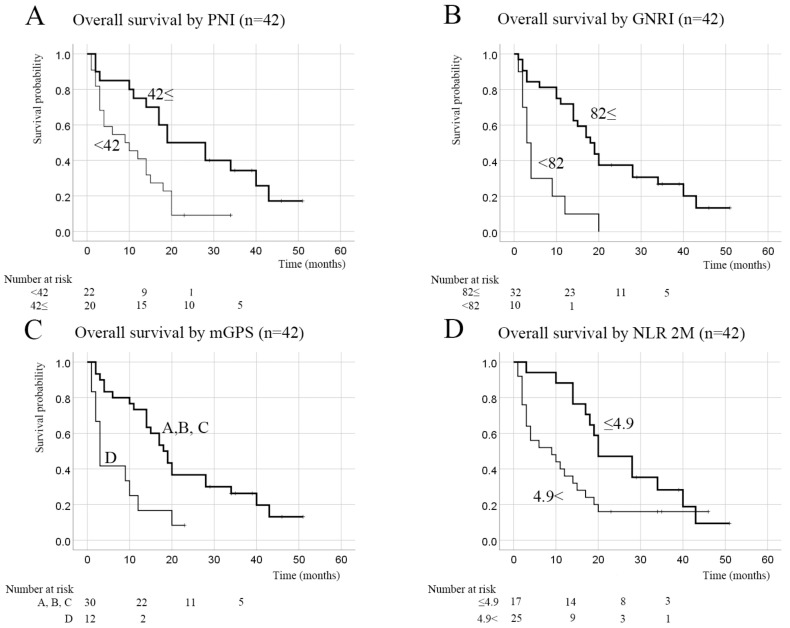
Kaplan–Meier curves for overall survival according to inflammatory and nutritional indexes in 42 patients. (**A**) PNI. Patients were categorized into two groups according to whether the PNI was <42 or ≥42. OS was significantly longer in the group with PNI ≥ 42 (*p* = 0.005). (**B**) GNRI. Patients who were well nourished (GNRI ≥ 82) had significantly longer survival times (*p* < 0.001). (**C**) mGPS. OS was significantly longer in patients with an mGPS of A, B, or C than in those with an mGPS of D (*p* = 0.002). (**D**) NLR at 2 months. OS was significantly longer in patients with an NLR < 4.9 at 8 weeks after starting nivolumab (*p* = 0.029). GNRI, geriatric nutritional risk index; mGPS, modified Glasgow prognostic score; NLR, neutrophil-to-lymphocyte ratio; OS, overall survival; PNI, prognostic nutritional index.

PNI was calculated using the serum albumin level and lymphocyte count at the start of the treatment with nivolumab. The mean and median PNI values were both 41.5. The OS was significantly longer in patients with PNI ≥ 42 than in those with PNI < 42 (*p* = 0.005, Figure 4A).

Nutritional status at the start of the treatment with nivolumab was evaluated using GNRI. GNRI was >98 (normal) in 14 patients, 92–98 (mildly poor nutrition) in 9 patients, 82–92 (moderately poor nutrition) in 9 patients, and <82 (severely poor nutrition) in 10 patients. Patients who were well nourished tended to have a longer survival time. The OS was significantly longer in patients with GNRI ≥ 82 (*p* < 0.001, Figure 4B). Although the ORR and DCR did not differ significantly according to whether GNRI was <82 or ≥82, patients with a value ≥ 82 tended to respond well to nivolumab (Table 3). Furthermore, patients with better nutritional status more frequently underwent subsequent treatment after nivolumab (*p* = 0.005, Table 3).

In terms of mGPS, there were 16 patients with A, 1 with B, 13 with C, and 12 with D (Table 4). The OS was significantly longer in patients with A, B, and C than in those with D (*p* = 0.002, Figure 4C).

### 3.4. Expression of PD-L1

The combined positive score (CPS) and tumor proportion score (TPS) were each divided into two groups according to whether the score was <1% or ≥1%. Twenty-seven patients had CPS and TPS ≥ 1%, and 22 had scores < 1% (Table 4). There was no significant difference in OS according to whether the CPS or TPS was ≥1% or <1%. Table 5 shows the treatment response according to PD-L1 expression. There was no significant difference in the DCR between the ≥1% and <1% TPS and CPS groups.

### 3.5. Occurrence of irAEs

Any irAE was observed in 11 (26.1%) of the 42 patients; grade 4 irAEs were observed in 2 patients, grade 3 irAEs in 5 patients, grade 2 irAEs in 8 patients, and grade 1 irAEs in 2 patients. Six patients had several distinct symptoms as a result of their irAEs. There was no significant difference in OS between patients with and without irAEs (Table 4). There were no significant differences in the occurrence of irAEs according to age, sex, PS, mGPS, GNRI, NLR, number of doses, or DCR (Table S1).

### 3.6. Multivariate Analysis of Clinical Parameters Potentially Affecting Overall Survival

Multivariate analysis was performed using the PS, GNRI, NLR at 2 months after nivolumab treatment, and number of nivolumab doses (Table 4). The PS, GNRI, and number of nivolumab doses were significantly associated with OS (*p* = 0.023, 0.003, and 0.05, respectively).

### 3.7. Analyses of SNPs

SNP analysis was performed in 35 of the 42 patients because all the blood samples had been used in other experiments in 7 cases. Eight (22.8%) of the 35 patients examined had some grade of irAE. Two SNPs in PD-L1, rs2282055 (intron) and rs4143815 (3′-untranslated region), were examined (Table 6). rs2282055 was T/T homozygous in 7 patients, T/G heterozygous in 15 patients, and G/G homozygous in 13 patients. The rs4143815 SNP was G/G homozygous in 11 cases, G/C heterozygous in 13 cases, and C/C homozygous in 11 cases. There were no significant differences in OS between patients with ancestral alleles (T/T in rs2282055 and G/G in rs4143815) and those with other alleles (Table 6). Patients with rs4143815 G/G and G/C alleles were significantly less likely to develop irAEs than those with C/C alleles (*p* = 0.006, Fisher’s exact test). Patients with T/T and T/G rs2282055 alleles also had a lower irAE rate (*p* = 0.03, Fisher’s exact test; Table 6).

## 4. Discussion

In this study, we retrospectively investigated patients with R/M-HNSCC with the aim of identifying biomarkers that could predict the response to ICI therapy and the occurrence of irAEs before starting treatment. We identified the following factors to be potential predictors based on previous experimental data and clinical trials: patient characteristics (age, sex, and PS), inflammatory and nutritional indicators (NLR, PNI, GNRI, and mGPS), occurrence of irAEs, immunohistological expression of PD-L1, and PD-L1 polymorphism. The PS and inflammatory and nutritional status were found to be the parameters most strongly associated with survival outcomes in patients treated with nivolumab. Crucially, these parameters can be rapidly determined through routine clinical measurements. 

A previous finding of a close relationship between PD-L1 expression and survival outcome [30] could not be confirmed in our study. There are several possible explanations for these inconsistent findings, including differences in the methods used to detect PD-L1 expression [31], the limited number of cases, and differences in tumor sites. The DAKO clone 28-8 pharmDX immunohistochemistry assays for PD-L1 scoring have been well developed [32], and a fair prognosis is expected in patients with ≥1% expression of PD-L1 who receive nivolumab [4]. Given that the PD-L1 antibody is unavailable for experimental studies in Japan, we used a substitute antibody for our histologic evaluation. The affinity for the PD-L1 antibody might have influenced the detection of PD-L1 expression in the present study [31]. The small number of participants in our study and the inclusion of various types of tumors may also have affected our results. Furthermore, in the present study, the samples used to determine PD-L1 expression were obtained before treatment. Considering that anticancer treatment induces the expression of PD-L1 [33], our sample collection time may also have affected our results. Therefore, we divided PD-L1 expression crudely into two categories, namely, <1% and ≥1%.

The survival outcomes during our long observation period included an ORR of 16.6% and a DCR of 50%. The OS rate was 59.5% at 1 year and 28.6% at 2 years, with a median OS of 15 months. These results were in line with or better than those previously reported [6,21]. In a multicenter study in Japan, in which 80% of subjects had a PS of 0 or 1, the median OS was 9.5 months, and the 1-year OS rate was 43.2% [6]. However, in the present study, more than 90% of patients had a PS of 0 or 1. The differences in general and nutritional conditions may have contributed to these differences in survival outcomes.

There have been several reports on the importance of the NLR when predicting survival outcomes in cancer therapy, including ICIs [34,35,36]. The NLR at 2 months after nivolumab treatment was related to survival in our univariate analysis. The NLR is affected by various conditions in cancer patients, including respiratory tract infection and inflammation in response to tumor growth itself, and patients may have a lower lymphocyte count and a weaker anti-tumor T-cell response if malnourished. The small number of samples in our study may have affected the association between the NLR and survival. Given that a dynamic change in the NLR in patients on ICI therapy is a prognostic marker [37], the NLR at 2 months following treatment with nivolumab may reflect the host response to nivolumab and might be a biomarker in the early phase of ICI treatment.

Alcohol and tobacco abuse are known to increase the risk of cancer [38] and are common in patients with HNC. Moreover, these lifestyle factors are associated with malnourishment in HNC patients. Nutritional management is crucial to avoid complications in patients undergoing major HNC surgery [39] and chemoradiotherapy [40] and to reduce the risk of cancer relapse [41]. The combination of pretreatment cachexia and weight loss during ICI therapy is associated with worse OS in patients with R/M-HNSCC [42]. Higher pretreatment PNI is also correlated with better prognosis in patients with HNC [20]. A low skeletal muscle index before treatment with an ICI has been found to have a negative impact on OS and PFS in patients with R/M-HNSCC [43]. In the present study, pretreatment PS and nutritional status (GNRI, mGPS, and PNI) were significant predictors of prognosis in patients treated with nivolumab. Our patients who received chemotherapy after 4 doses of nivolumab had better OS and PFS-2. After ICI treatment, salvage chemotherapy regimens, especially PTX + Cet, were found to achieve better prognoses in patients with a lower C-reactive protein level or NLR at induction than in those with a higher C-reactive protein level or NLR [44]. The PTX + Cet [45] regimen was the most frequently used second-line treatment. The findings of our present study and those in previous reports suggest that patients with a good PS and adequate nutrition can receive salvage chemotherapy after treatment with nivolumab.

Structural polymorphisms in the 3′ untranslated region of PD-L1 cause the overexpression of PD-L1 and affect the prognosis in patients with cancer. More than 1000 SNPs in the 3′ untranslated region of PD-L1 have been registered with the NCBI (National Center for Biotechnology Information). Three SNPs—rs2282055, rs4143815, and rs4742098—have a frequency of more than 10%. SNPs in the 3′ untranslated region of PD-L1 have been reported to affect the binding of miRNA and control the expression of PD-L1 [15], and they may affect the prognosis of cancer [16]. In the present study, there was no significant relationship between rs2282055 and rs4143815 and the survival outcomes in patients treated with nivolumab. A previous report demonstrated that nivolumab had fair therapeutic effects in patients with irAEs [46]. However, in the present study, there was no close relationship between the occurrence of irAEs and treatment effects. Considering that general, inflammatory, and nutritional conditions are important indicators of survival outcomes, it is possible that our sample size was too small and may have masked the effect of SNPs and irAEs on survival outcomes.

One of the novel findings in our study was the relationship between SNPs (rs2282055 and rs4143815) and the occurrence of irAEs. Our patients with ancestral alleles (T/T in rs2282055 and G/G in rs4143815) did not have irAEs, although the number of cases was limited. It has been suggested that patients with these SNPs in PD-L1 are at higher risk of cancer and that survival in these patients can be improved by anti-PD-L1 therapy. The 1000 Genomes Project Phase 3 has shown that the allele frequencies are 35% T and 65% G for rs2282055 and 46.2% G and 53.8% C for rs4143815 in Japan, while they are 71% T and 29% G for rs2282055 and 67.0% G and 33.0% C for rs4143815 in Europe. Therefore, the frequencies of SNPs in PD-L1 may vary according to ethnicity. The exact mechanism via which SNPs affect the risk of irAEs is unclear, and there is limited information on the relationship between germline SNPs and irAEs [47]. Although the association between the toxicities of nivolumab and SNPs as contributors to PD-1-directed T-cell responses has been extensively investigated, SNPs have not been clinically implicated in the toxicity of nivolumab [47]. Seven SNPs in 4 genes, PDCD1, PTPN11, ZAP70, and IFNG, were identified in 322 patients with NSCLC treated with nivolumab. Germline DNAs were genotyped using a custom panel of 86 preselected immunogenetically related genes in 94 consecutive patients with advanced cancer treated with anti-PD-1/PD-L1 checkpoint inhibitors. Toxicity was linked to target-related gene SNPs, including PD-L1, UNG, IFNW1, CTLA4, and IFNL4 genes [9]. The relationship between SNPs and irAEs has not been established. PD-L1 is expressed not only on tumor cells but also on host immune-related cells. Therefore, treatment with nivolumab can elicit an immune reaction in the host. The results of our present study shed some light on predicting the occurrence of irAEs. However, more extensive investigations, including those of ethnicity and the possibility of a mutual relationship of multiple SNPs, are needed to clarify the role of SNPs in the development of irAEs in patients receiving anti-PD-L1 therapy.

A multicenter real-world study in Japan [6] found that any-grade irAEs were observed in 44 of 256 patients (17.2%). In our present study, the occurrence rate of irAEs (26.1%) was in line with that previous report. Patients with T/T and T/G rs2282055 alleles and G/G and G/C rs4143815 alleles were significantly less likely to develop irAEs than patients with G/G rs2282055 alleles and C/C rs4143815 alleles, respectively. Although the occurrence rates of irAEs differed significantly between patients with these alleles, the sample size in the present study was small. To confirm these results, a sample size calculation indicates that we would need approximately 70 patients for PD-L1 SNP analysis. Thus, further analysis is needed to address this issue.

## 5. Conclusions

Anti-PD-1 antibodies are beneficial for patients with R/M-HNSCC in terms of prolonged survival, but biomarkers are needed that can predict the response to ICI therapy and the occurrence of irAEs before starting treatment. The present study identified performance status and inflammatory and nutritional status as significant indicators of survival outcomes. These parameters could be rapidly determined through routine clinical measurements. The occurrence of irAEs was less frequent in patients with ancestral alleles in PD-L1 polymorphisms (rs2282055 and rs4143815). The results of this study shed some light on predicting survival benefits and the occurrence of irAEs for patients with R/M-HNSCC.

## Figures and Tables

**Table 1:** Clinical characteristics of the 42 patients.

Indicator	Category	*n*	%
Sex	Male	36	85.7
	Female	6	14.3
Primary site	Sinonasal cavity	1	2.4
	Oral cavity	9	21.4
	Nasopharynx	2	4.8
	Oropharynx	13	31.0
	HPV-positive	7	
	HPV-negative	6	
	Hypopharynx	14	33.3
	Larynx	1	2.4
	Salivary gland	1	2.4
	Primary unknown	1	2.4
Performance status	0	29	69.0
	1	10	23.8
	≥2	3	7.1
Lines of nivolumab after primary treatment			
	First	12	28.6
	Second	14	33.3
	Third	10	23.8
	Fourth or later	6	14.3
Tumor location at first nivolumab administration			
	Local recurrence/progression	14	33.3
	Regional recurrence/progression	10	23.8
	Distant metastasis	31	73.8

HPV, human papillomavirus; *n*, number of subjects.

**Table 2:** Treatment course in 42 study participants.

Treatment Course		*n*
Continued nivolumab		1
Cause	CR	1
Switched to other treatment		18
Response	CR	1
	PR	3
	SD	7
	PD	7
Discontinuation of all treatment		23
Cause	PD	13
	Poor general condition	9
	CR	1

CR, complete response; *n*, number of subjects; PD, progressive disease; PR, partial response; SD, stable disease.

**Table 3:** Nutritional status and treatment response.

			GNRI Score		
		*n* (%)	<82	≥82	*p*-Value
BOR					
	CR	3 (7.1)	0	3	
	PR	4 (9.5)	1	3	
	SD	14 (33.3)	3	11	
	PD	21 (50.0)	8	13	
ORR		7 (16.7)	1	6	0.65
DCR		21 (50.0)	4	17	0.3
Next treatment		18 (42.9)	1	17	0.005

BOR, best overall response; CR, complete response; DCR, disease control rate; GNRI, geriatric nutritional index; *n*, number of subjects; ORR, overall response rate; PD, progressive disease; PR, partial response; SD, stable disease.

**Table 4:** Demographic and clinical characteristics and prognosis in the 42 patients.

Variable		Cases, *n*	Mean OS (Days)	Univariate Analysis (*p*-Value)	Multivariate Analysis (*p*-Value)	HR (95% CI)
Age	Age, years					
	≤60	22	634	0.546		
	>60	20	536			
Sex	Sex					
	Male	36	619	0.255		
	Female	6	434			
PS						
	0, 1	39	618	0.048	0.013	0.193 (0.053–0.712)
	≥2	3	234		Reference	
NLR						
	≤4.9	21	686	0.222		
	>4.9	21	492			
NLR 2 months after nivolumab						
	≤4.9	17	813	0.029		
	>4.9	25	437			
Eosinophil count						
	≤170	25	549	0.713		
	>170	17	631			
Enteral nutrition				0.395		
	Yes	6	473			
	No	36	616			
PNI						
	<42	22	361	0.004		
	≥42	20	816			
GNRI						
	>98	14	892			
	92–98	9	552			
	82–92	9	594			
	<82	10	200			
	≥82	32	712	<0.001	0.002	0.249 (0.105–0.592)
	<82	10	200		Reference	
mGPS						
	A	16	791			
	B	1	129			
	C	13	648			
	D	12	244			
	A, B, C	30	710	0.002		
	D	12	244			
CPS						
	<1	15	603	0.752		
	≥1	27	573			
TPS						
	<1	20	622	0.491		
	≥1	22	543			
irAEs						
	Present	11	543	0.355	0.302	
	Absent	31	697		Reference	
Nivolumab doses, *n*						
	≤4	16	266	<0.001	Reference	
	>4	26	769		0.05	2.457 (1.000–6.040)

CI, confidence interval; CPS, combined positive score; GNRI, geriatric nutritional risk index; HR, hazard ratio; irAEs, immune-related adverse events; mGPS, modified Glasgow prognostic score; *n*, number of subjects; NLR, neutrophil-to-lymphocyte ratio; OS, overall survival; PNI, prognostic nutritional index; TPS, tumor proportion score.

**Table 5:** PD-L1 expression and survival outcomes.

PD-L1 Expression		CR, PR, SD	PD	DCR	*p*-Value
TPS	<1%	11	9	55.0%	0.537
	≥1%	10	12	45.5%	
CPS	<1%	9	6	60.0%	0.334
	≥1%	12	15	44.4%	

CPS, combined positive score; CR, complete response; DCR, disease control rate; OS, overall survival; PD, progressive disease; PD-L1, programmed cell death-1 ligand; PR, partial response; SD, stable disease; TPS, tumor proportion score.

**Table 6:** Survival outcomes and occurrence of irAEs according to PD-L1 single nucleotide polymorphism.

	Cases, *n*	Mean OS (Days)	Median OS (Days)	*p*-Value	irAEs		*p*-Value
					Absent	Present	
rs2282055							
T/T	7	796	627	0.344	7	0	TT, TG vs. GG
T/G	15	565	531		13	2	0.03
G/G	13	627	470		7	6	
rs4143815							
G/G	11	573	451	0.728	11	0	GG, GC vs. CC
G/C	13	605	565		11	2	0.006
C/C	11	743	622		5	6	

irAEs, immune-related adverse events; OS, overall survival.

## Data Availability

The datasets generated and/or analyzed during the present study have not been made publicly available. However, data can be made available from the corresponding author upon reasonable request.

## Supplementary Materials

The following supporting information can be downloaded at: https://www.mdpi.com/article/10.3390/curroncol30060410/s1, Table S1: Immune-related adverse events and clinical features (42 patients).
