# Supplementary material for: Biomarkers for Predicting Anti-Programmed Cell Death-1 Antibody Treatment Effects in Head and Neck Cancer
Source: Curr Oncol. 2023 Jun 2;30(6):5409–24. doi: 10.3390/curroncol30060410 (PMC10297315; doi:10.3390/curroncol30060410)
Supplement: Supplementary file 1 [file curroncol-30-00410-s001.zip › curroncol-2391477-supplementary.pdf]

Supplementary Materials

Biomarkers for Predicting Anti-Programmed Cell Death-1 Antibody Treatment Effects in Head and Neck Cancer

Katsunori Tanaka, Hitoshi Hirakawa, Mikio Suzuki *, Teruyuki Higa, Shinya Agena, Narumi Hasegawa, Junko Kawakami, Masatomo Toyama, Tomoyo Higa, Hidetoshi Kinjyo, Norimoto Kise, Shunsuke Kondo, Hiroyuki Maeda and Taro Ikegami

Department of Otorhinolaryngology, Head and Neck Surgery, Graduate School of Medicine, University of the Ryukyus, 207 Uehara, Nishihara-cho, Nakagami-gun, Okinawa 903-0215, Japan; mizuki0415@gmail.com (K.T.); aoi23@med.u-ryukyu.ac.jp (H.H.); tellurteru@yahoo.co.jp (T.H.); harugen3@yahoo.co.jp (S.A.); puyoraer99110@gmail.com (M.T.); tomoyo_12_26@hotmail.co.jp (T.H.); hidechanman223@yahoo.co.jp (H.K.); norimoto7@gmail.com (N.K.); kouhouiinn@yahoo.co.jp (S.K.); maeidahiroyuki@yahoo.co.jp (H.M.); ikegami@med.u-ryukyu.ac.jp (T.I.)

* Correspondence: suzuki@med.u-ryukyu.ac.jp; Tel.: +81-895-1183

Table S1. Clinical characteristics and immune-related adverse events in 42 patients with recurrent or metastatic head and neck cancer.

	irAE		
**Variable**	**Absent**	**Present**	***p*-Value**
Age, years			
≤60	15	3	0.224
<60	16	8	
Sex			
Male	27	9	0.667
Female	4	2	
ECOG PS			
0, 1	29	9	
≥2	2	2	
mGPS			
0, 1	23	7	0.5
2	8	4	
GNRI			
≥92	19	4	0.154
<92	12	7	
NLR when starting nivolumab			
<4.9	15	6	0.726
≥4.9	16	5	
Doses, n			
≤4	12	4	0.891
>4	19	7	
DCR			
CR, PR, SD	16	5	0.726
PD	15	6	

CR, complete response; DCR, disease control rate; GNRI, geriatric nutritional risk index; irAE, immune-related adverse event; mGPS, modified Glasgow prognostic score; NLR, neutrophil-to-lymphocyte ratio; PD, progressive disease; PR, partial response; SD, stable disease.
